# Barrett's Oesophagus in an Achalasia Patient: Immunological Analysis and Comparison with a Group of Achalasia Patients

**DOI:** 10.1155/2016/5681590

**Published:** 2016-09-26

**Authors:** Samuel Torres-Landa, Janette Furuzawa-Carballeda, Enrique Coss-Adame, Miguel A. Valdovinos, Edgar Alejandro-Medrano, Bárbara Ramos-Ávalos, Braulio Martínez-Benítez, Gonzalo Torres-Villalobos

**Affiliations:** ^1^Department of Experimental Surgery, Instituto Nacional de Ciencias Médicas y Nutrición Salvador Zubirán, Vasco de Quiroga No. 15, Colonia Belisario Domínguez Sección XVI, 14080 Mexico City, Mexico; ^2^Department of Immunology and Rheumatology, Instituto Nacional de Ciencias Médicas y Nutrición Salvador Zubirán, Vasco de Quiroga No. 15, Colonia Belisario Domínguez Sección XVI, 14080 Mexico City, Mexico; ^3^Department of Gastroenterology, Instituto Nacional de Ciencias Médicas y Nutrición Salvador Zubirán, Vasco de Quiroga No. 15, Colonia Belisario Domínguez Sección XVI, 14080 Mexico City, Mexico; ^4^Department of Pathology, Instituto Nacional de Ciencias Médicas y Nutrición Salvador Zubirán, Vasco de Quiroga No. 15, Colonia Belisario Domínguez Sección XVI, 14080 Mexico City, Mexico

## Abstract

The aim of the study was to characterize the presence of diverse CD4 and CD8 T cell subsets and regulatory cells in peripheral blood and lower oesophageal sphincter (LES) from a young patient with BE/achalasia without treatment versus achalasia group. In order to characterize the circulating cells in this patient, a cytometric analysis was performed. LES tissue was evaluated by double-immunostaining procedure. Five healthy blood donors, 5 type achalasia patients, and 5 oesophagus tissue samples (gastrooesophageal junction) from transplant donors were included as control groups. A conspicuous systemic inflammation was determined in BE/achalasia patient and achalasia versus healthy volunteer group. Nonetheless, a predominance of Th22, Th2, IFN-*α*-producing T cells, Tregs, Bregs, and pDCregs was observed in BE/achalasia patient versus achalasia group. A low percentage of Th1 subset in BE/achalasia versus achalasia group was determined. A noticeable increase in tissue of Th22, Th17, Th2, Tregs, Bregs, and pDCregs was observed in BE/achalasia versus achalasia group. Th1 subset was lower in the BE/achalasia patient versus achalasia group. This study suggests that inflammation is a possible factor in the pathogenesis of BE/achalasia. Further research needs to be performed to understand the specific cause of the correlation between BE and achalasia.

## 1. Background

Idiopathic achalasia is a primary oesophageal motor disorder characterized by the loss of oesophageal peristalsis and incomplete relaxation of the lower oesophageal sphincter (LES). The annual incidence and prevalence are 1 case per 100,000 and 10 in every 1,000,000 habitants, respectively [1]. Recent studies have suggested that achalasia has an important local and systemic autoimmune inflammatory response, with a high prevalence of specific autoantibodies against the myenteric plexus, and it has been associated with the presence of Herpes simplex virus type 1 [2]. Clinical manifestations include dysphagia, regurgitation, retrosternal chest pain, weight loss, and heartburn. Diagnosis is established by manometric studies and complementary tests like barium esophagram and esophagogastroduodenoscopy [1].

Barrett's oesophagus (BE) is a disease in which the normal distal squamous epithelial lining is replaced by metaplastic columnar epithelium that can be suspected by endoscopy and confirmed by histopathology [3]. BE/achalasia as cooccurring disease has been reported in up to 14% following treatment of achalasia [4]. However, in untreated patients this cooccurring finding is a rare phenomenon, and to our knowledge there are only 4 studies that have reported this association [5–8].

It is important to mention that the 4 patients previously reported were older (≥40 years), the age being one of the major risk factors for BE due to the long natural history of the disease. In this case report we present the first report of immunological analysis of BE/achalasia in a young patient with no previous surgical or endoscopic treatment, diagnosed with BE.

## 2. Case Presentation

**Figure 1:**
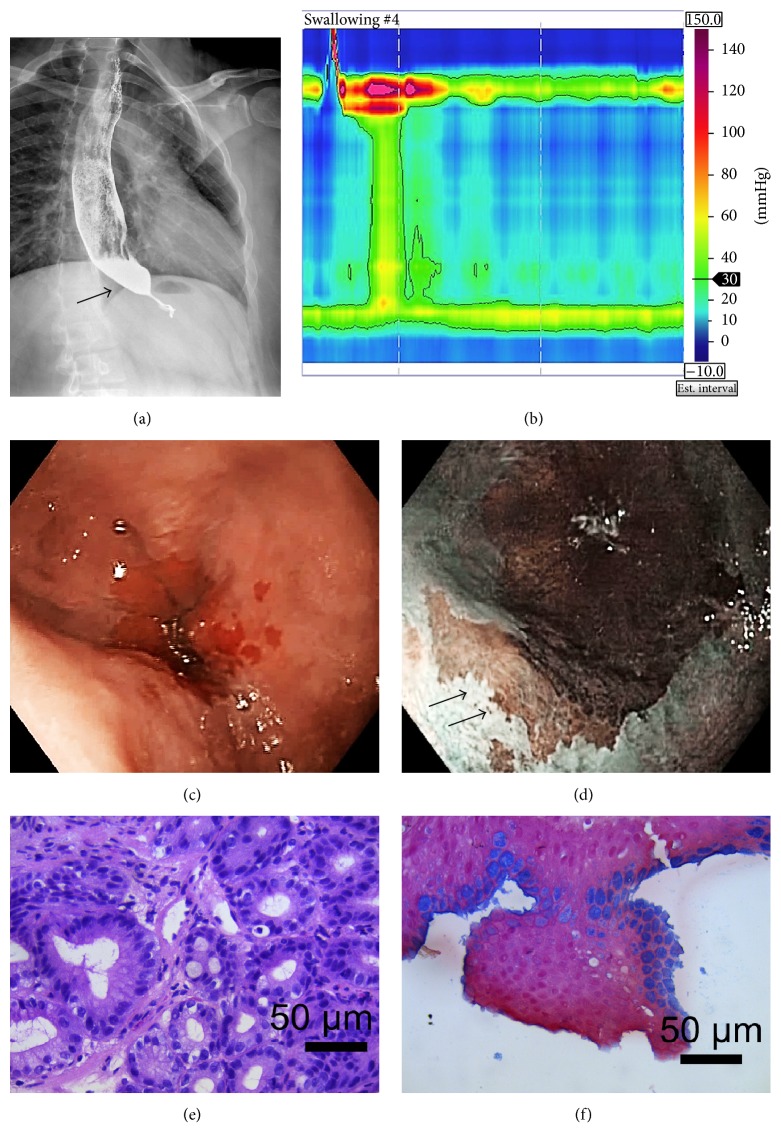
(a) Barium oesophagram that shows dilatation and bird beak sign (arrow). (b) High resolution manometry that shows abnormal relaxation of lower oesophageal sphincter and aperistalsis with panesophageal pressurization in 40% of swallows, corresponding with type II achalasia. (c) Upper endoscopy shows dilated esophagus with mucosal changes consistent with C0 M1 Barrett's oesophagus. (d) Arrows indicate margins of “tongues” of Barrett's metaplasia. (e) Metaplastic columnar epithelium. (f) Photomicrograph that highlights the metaplasia. Original magnification in high field power (100x).

This case is of a 28-year-old man with a previous history of chronic hypertension, 12 years of tobacco consumption, and 7 years of alcohol consumption every 2 weeks, without any family history of BE. He was referred to our hospital after 1 year of progressive oesophageal dysphagia, associated with regurgitation, retrosternal chest pain, and weight loss of 7 kg. Physical examination was unremarkable, and BMI was 28.37 at the moment of referral (BMI of 30.82 prior to the disease). A barium swallow showed oesophageal dilatation and bird beak sign (Figure 1(a)). A high resolution manometry (HRM) (Figure 1(b)) and panendoscopy were performed. The HRM showed an elevated IRP value of 31.2 mmHg, abnormal relaxation of lower oesophageal sphincter (LES), and aperistalsis with panoesophageal pressurization in >20% of swallows, corresponding with type II achalasia. Hiatal hernia was not reported. Unexpectedly during the endoscopy procedure, irregular Z-line was seen with tong-like protrusions suspicious of BE that was classified as C 0 M1 accordingly with the Prague Classification (Figures 1(c) and 1(d)). Biopsies of suspicious areas were taken and revised by two expert pathologists that reported metaplastic columnar epithelium (Barrett's oesophagus) without dysplasia (Figures 1(e) and 1(f)). The oesophageal 24 h impedance-pH monitoring showed normal acid exposure with 0.2% of pH < 4 and a DeMeester score of 1.1. The patient underwent Heller's myotomy with biopsy of the muscular layer of the oesophagus and Dor fundoplication in November 2015. Transsurgical endoscopy was negative for leakages. Within the first 24 hours the patient developed systemic inflammatory response syndrome. A water-soluble study was performed, which reported a possible leak from the myotomy site. CT scan reported pneumomediastinum without a visible leakage. He underwent a laparoscopic exploration without a visible site of leakage and negative cultures. The patient was treated without any other intervention, only with fasting, and was discharged without any further complications.

### 2.1. Ethical Considerations

The study was approved by the ethical medical committee in our institution (reference number 1522) and it was according to the principles expressed in the Declaration of Helsinki, 1989. Only patients who gave a written informed consent were recruited for this study. Each participant gave a written consent to publish their individual data.

## 3. Discussion

### 3.1. Barrett's Oesophagus and Achalasia

Although achalasia has been related to BE after surgical procedure [4], there is no clear explanation of the cause of this finding in a young patient before myotomy. Nonetheless, it is known that BE has been correlated to acid exposure due to gastric oesophageal reflux disease (GERD) [9]. One of the possibilities of this rare finding could be a chronic acid exposure secondary to GERD prior to the developing of achalasia. Another one could be that, in achalasia patients, food retention and its fermentation lower the pH due to lactic acid production, allowing chronic irritation against the oesophageal epithelium [9]. Although these two hypotheses cannot be confirmed, it is a fact that our patient had many of the risk factors (grade of evidence: II and IIa) for BE [3] including sex, heartburn, smoking, alcohol consumption, and BMI > 30.

### 3.2. Immunophenotyping of Circulating Regulatory Cell and CD4 Effector T Cell Subpopulations by Flow Cytometry

In a recent publication, our group [2] determined the peripheral blood immunophenotypes of different subtypes of achalasia. In order to characterize the peripheral blood compartment in this patient, we proceeded to follow the same methodology [2]. A group of 5 healthy blood donors and 5 type II achalasia patients, specifically sampled for this study, were included as control groups. A sample of venous blood (10 mL) was obtained from each subject. Peripheral blood mononuclear cells (PBMCs) were isolated by gradient centrifugation on Lymphoprep (Axis-Shield PoC AS, Oslo, Norway). Th22 (CD3^+^/CD4^+^/CD161^−^/IL-22^+^), Th17 (CD3^+^/CD4^+^/CD161^+^/IL-17A^+^), Th2 (CD3^+^/CD4^+^/CD25^+^/IL-4^+^), Th1 (CD3^+^/CD4^+^/CD25^+^/IFN-*γ* ^+^), IFN-*α*-producing T cells (CD3^+^/CD4^+^/CD25^+^/IFN-*α* ^+^ and CD3^+^/CD8^+^/CD28^+^/IFN-*α* ^+^), regulatory T cells (CD3^+^/CD4^+^/CD25^hi^/Foxp3^+^, CD3^+^/CD4^+^/CD25^hi^/IL-35^+^, CD3^+^/CD8^+^/CD28^−^/Foxp3^+^, and CD3^+^/CD8^+^/CD28^−^/IL-35^+^), regulatory B cells (CD3^+^/CD19^+^/CD38^hi^/IL-10^+^ and CD3^+^/CD19^+^/CD38^hi^/IL-35^+^), and regulatory plasmacytoid dendritic cells (pDCs; CD123^hi^/CD196^+^/IDO^+^) were determined. In the cytometric analysis, a conspicuous systemic inflammation was determined in BE/achalasia patient and achalasia group compared with healthy volunteer group (Table 1). Nonetheless, a predominance of Th22, Th2, IFN-*α*-producing T cells, Tregs, Bregs, and pDCregs was observed in BE/achalasia patient compared with achalasia group. In contrast a low percentage of Th1 subset in BE/achalasia versus achalasia patient group was determined (Table 1). Th17 profile was similar in our BE/achalasia patient compared with achalasia group (Table 1).

### 3.3. Characterization of Tissue Regulatory Cell and CD4 Effector Cell Subsets by Immunohistochemistry

**Figure 2:**
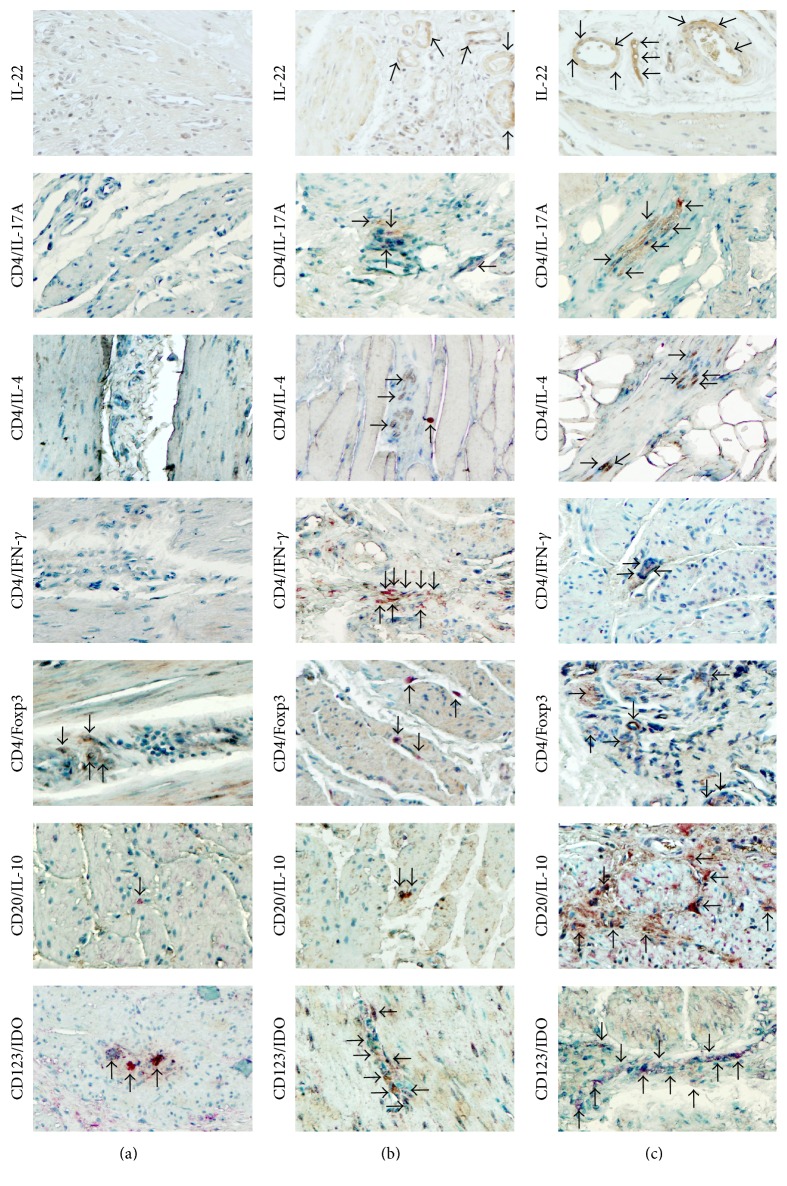
CD4 effector T cells and regulatory cells in lower oesophageal sphincter. Representative photomicrograph from control (healthy donor, (a)), type II achalasia (b), and Barrett's oesophagus/type II achalasia patient (c). Arrows depict immunoreactive cells. Original magnification was ×320.

In order to determine the characteristics of the cellular infiltrate, regulatory cells, and interleukin expression present at the lower oesophageal muscle, tissue was stained with specific monoclonal antibodies. Five type II achalasia patients and 5 oesophagus tissue samples (gastrooesophageal junction) from transplant donors were included as a control tissue (Figure 2). Thus, the subpopulation of IL-22-producing cells, CD4^+^/IL-17A^+^-, CD4^+^/IL-4^+^-, CD4^+^/IFN-*γ* ^+^-expressing T cells, CD25^+^/Foxp3^+^ regulatory T cells, CD123^+^/IDO^+^ pDCs, and CD20^+^/IL-10^+^-producing B cells was determined with a simultaneous detection using a Multiview (mouse-HRP/rabbit-AP) immunohistochemistry kit (Enzo Life Sciences, Inc., Farmingdale, NY, USA) [2]. Immunohistochemistry findings showed greater amounts of inflammatory and regulatory cells in BE/achalasia patient and achalasia group compared with control group (Table 1). On the other hand, a noticeable increase in Th22, Th17, Th2, Tregs, Bregs, and pDCregs was observed in BE/achalasia compared to achalasia patient group (Table 1, Figure 2). Th1 subset was lower in the BE/achalasia patient compared with the achalasia group (Table 1, Figure 2).

### 3.4. Appropriate Treatment and Surveillance

One of the main concerns regarding this patient condition is the posterior risk of developing dysplasia. It has been reported that, due to the high acid exposure presented after Heller's myotomy, a partial fundoplication is now recommended to reduce gastrooesophageal reflux [10]. Although Dor/Toupet fundoplication is more commonly performed on these patients, a significant difference between both partial fundoplications acting as acid barrier has not been reported [11]. The patient underwent an appropriate treatment for his condition although a close surveillance with symptoms, acid exposure, and upper endoscopy could be needed.

Our data suggest that patients with BE/achalasia could have higher inflammation and a higher risk of oesophageal perforation compared with achalasia patients. Moreover, inflammation is considered as the connection of Barrett's carcinogenesis. Key mediators of inflammation in BE have been previously described and include proinflammatory cytokines, chemokines, reactive oxygen species, prostaglandins, and microRNAs [12]. Moons et al. have also demonstrated an increase of Th2 effector cells in BE tissue [13]. Moreover, in serum from BE patients a strong positive association with high level of IL-12p70, IL-8, and leptin and a negative association with IL-10 and IL-1*β* have been determined. Meanwhile there were no differences between serum controls and BE patients in levels of IFN-*γ*, TNF-*α*, adiponectin, or insulin [14]. In the BE/achalasia patient we have shown that Th22, Th17, and Th2 but not Th1 profile interplay at tissue and peripheral cells in BE/achalasia patient. In addition to the presence of proinflammatory cells there is an increase of Treg, IL-10-producing B cell, and pDCreg cell percentage suggesting that proinflammatory and regulatory balance favours the former with the failure of the latter in maintaining homeostasis and conducting more vigorous tissue damage. However this study suggests that inflammation is a possible factor in the pathogenesis of BE/achalasia with the concomitant use of immunosuppressive drugs as probable future treatment for this pathology. It is relevant to highlight the need for a close follow-up to prevent further complications.

## 4. Conclusion

In conclusion, our preliminary results deserve to be studied in depth to appraise the clinical relevance of these findings. It is also necessary to clarify whether the association of BE and achalasia is an epiphenomenon or might share common pathophysiological pathways.

## Figures and Tables

**Table 1:** Analysis of peripheral blood and tissue immunophenotypes in the present case compared to 5 achalasia patients and 5 control subjects.

	Healthy donors (*n* = 5)	Type II achalasia (*n* = 5)	Patient
	Blood		
*Peripheral blood immunophenotypes*			
*CD3* ^*+*^ */CD4* ^*+*^ */CD161* ^−^ */IL-22* ^*+*^ * (%)*			
Mean ± SD	1.95 ± 0.53	6.58 ± 2.56	**7.72**
Median	1.79	4.90	
Range	1.39–2.55	2.79–16.70	
*CD3* ^*+*^ */CD4* ^*+*^ */CD161* ^*+*^ */IL-17A* ^*+*^ * (%)*			
Mean ± SD	1.96 ± 0.80	5.62 ± 1.73	5.42
Median	1.64	4.95	
Range	1.40–3.35	2.72–12.30	
*CD3* ^*+*^ */CD4* ^*+*^ */CD25* ^*+*^ */IL-4* ^*+*^ * (%)*			
Mean ± SD	1.44 ± 0.65	3.23 ± 0.70	**4.19**
Median	1.36	3.76	
Range	0.52–2.09	1.55–4.99	
*CD3* ^*+*^ */CD4* ^*+*^ */CD25* ^*+*^ */IFN-γ* ^*+*^ * (%)*			
Mean ± SD	1.76 ± 0.97	4.18 ± 1.31	3.6
Median	1.91	3.43	
Range	0.29–2.87	2.15–9.30	
*CD3* ^*+*^ */CD4* ^*+*^ */CD25* ^*+*^ */IFN-α* ^*+*^ * (%)*			
Mean ± SD	2.26 ± 1.2	4.47 ± 0.85	**5.08**
Median	1.81	3.73	
Range	1.31–4.35	2.11–6.80	
*CD3* ^*+*^ */CD8* ^*+*^ */CD28* ^*+*^ */IFN-α* ^*+*^ * (%)*			
Mean ± SD	3.05 ± 1.97	3.99 ± 0.47	**5.87**
Median	2.82	4.28	
Range	1.48–6.39	2.54–5.27	
*CD3* ^*+*^ */CD4* ^*+*^ */CD25* ^*hi*^ */Foxp3* ^*+*^ * (%)*			
Mean ± SD	5.16 ± 0.78	7.21 ± 0.45	**10.1**
Median	5.05	7.32	
Range	4.00–6.03	5.64–8.35	
*CD3* ^*+*^ */CD4* ^*+*^ */CD25* ^*hi*^ */IL-35* ^*+*^ * (%)*			
Mean ± SD	3.60 ± 0.98	4.08 ± 0.44	9.96
Median	3.64	4.28	
Range	2.50–5.11	2.39–4.88	
*CD3* ^*+*^ */CD8* ^*+*^ */CD28* ^−^ */Foxp3* ^*+*^ * (%)*			
Mean ± SD	3.82 ± 0.71	5.39 ± 0.28	**6.72**
Median	3.95	5.46	
Range	3.01–4.77	4.42–6.06	
*CD3* ^*+*^ */CD8* ^*+*^ */CD28* ^−^ */IL-35* ^*+*^ * (%)*			
Mean ± SD	3.37 ± 0.77	4.53 ± 1.28	**12.4**
Median	3.69	3.02	
Range	2.19–4.01	2.12–9.09	
*CD3* ^*+*^ */CD19* ^*+*^ */CD38* ^*+*^ */IL-10* ^*+*^ * (%)*			
Mean ± SD	6.27 ± 1.07	6.99 ± 0.58	**14.1**
Median	6.08	6.50	
Range	5.07–7.81	4.88–8.09	
*CD3* ^*+*^ */CD19* ^*+*^ */CD38* ^*+*^ */IL-35* ^*+*^ * (%)*			
Mean ± SD	5.95 ± 0.45	4.31 ± 0.89	**19.2**
Median	5.88	3.90	
Range	5.58–6.70	2.20–7.60	
*CD123* ^*+*^ */CD196* ^*+*^ */IDO* ^*+*^ * (%)*			
Mean ± SD	11.84 ± 1.36	16.82 ± 1.66	**21.2**
Median	11.50	18.70	
Range	10.30–14.00	10.80–19.47	

	Tissue		
*Tissue immunophenotypes*			
*IL-22-expressing cells (%)*			
Mean ± SD	3.83 ± 1.83	17.83 ± 1.47	**23.5**
Median	4.00	17.50	
Range	2.00–7.00	16.00–20.00	
*IL-17A-expressing CD4 T cells (%)*			
Mean ± SD	1.00 ± 0.68	8.33 ± 1.75	**17.00**
Median	0.00	7.50	
Range	0.00–4.00	7.00–11.00	
*IL-4-expressing CD4 T cells (%)*			
Mean ± SD	1.33 ± 0.33	6.33 ± 0.75	**14.00**
Median	1.50	7.00	
Range	0.00–2.00	4.00–9.00	
*IFN-γ-expressing CD4 T cells (%)*			
Mean ± SD	1.50 ± 0.67	15.0 ± 1.27	10.00
Median	1.00	14.5	
Range	0.00–4.00	14.00–17.00	
*Foxp3-expressing CD25 T cells (%)*			
Mean ± SD	4.33 ± 0.33	7.83 ± 0.48	**11.50**
Median	4.50	8.00	
Range	3.00–5.00	6.00–9.00	
*IL-10-expressing CD20 B cells (%)*			
Mean ± SD	1.83 ± 1.31	5.33 ± 0.49	**7.5**
Median	2.00	5.50	
Range	1.00–3.00	4.00–7.00	
*IDO-expressing CD123 cells (%)*			
Mean ± SD	3.67 ± 0.82	6.67 ± 0.33	**8.5**
Median	3.50	6.50	
Range	3.00–5.00	6.00–8.00	

## Abbreviations

AP: Alkaline phosphatase
BE: Barrett's oesophagus
BMI: Body Mass Index
GERD: Gastric oesophageal reflux disease
HRM: High resolution manometry
HRP: Horseradish peroxidase
IFN: Interferon
IL: Interleukin
LES: Lower oesophageal sphincter
PBMCs: Peripheral blood mononuclear cells
pDCreg: Regulatory plasmacytoid dendritic cell
Th: T helper cell
TNF: Tumour necrosis factor
Treg: Regulatory T cell.
